# Effects of temperature, rainfall, and El Niño Southern Oscillations on dengue-like-illness incidence in Solomon Islands

**DOI:** 10.1186/s12879-023-08188-x

**Published:** 2023-04-06

**Authors:** Gerry Andhikaputra, Yu-Han Lin, Yu-Chun Wang

**Affiliations:** 1 Department of Environmental Engineering, College of Engineering, Chung Yuan Christian University, 200 Chung-Pei Road, Zhongli, 320 Taiwan; 2 Research Center for Environmental Changes, Academia Sinica, 128 Academia Road, Section 2, Nankang, Taipei 11529 Taiwan

**Keywords:** Dengue-like-illness, ENSO, Multivariate analysis, Solomon islands

## Abstract

**Background:**

This study investigated associations between climate variables (average temperature and cumulative rainfall), and El Niño Southern Oscillation (ENSO) and dengue-like-illness (DLI) incidence in two provinces (Western and Guadalcanal Provinces) in Solomon Islands (SI).

**Methods:**

Weekly DLI and meteorological data were obtained from the Ministry of Health and Medical Services SI and the Ministry of Environment, Climate Change, Disaster Management and Meteorology from 2015 to 2018, respectively. We used negative binomial generalized estimating equations to assess the effects of climate variables up to a lag of 2 months and ENSO on DLI incidence in SI.

**Results:**

We captured an upsurge in DLI trend between August 2016 and April 2017. We found the effects of average temperature on DLI in Guadalcanal Province at lag of one month (IRR: 2.186, 95% CI: 1.094–4.368). Rainfall had minor but consistent effect in all provinces. La Niña associated with increased DLI risks in Guadalcanal Province (IRR: 4.537, 95% CI: 2.042–10.083), whereas El Niño associated with risk reduction ranging from 72.8% to 76.7% in both provinces.

**Conclusions:**

Owing to the effects of climate variability and ENSO on DLI, defining suitable and sustainable measures to control dengue transmission and enhancing community resilience against climate change in low- and middle-developed countries are important.

**Supplementary Information:**

The online version contains supplementary material available at 10.1186/s12879-023-08188-x.

## Introduction

Dengue is an acute vector-borne disease transmitted between humans by single-strand positive-sense RNA viruses (DENV-1–DENV-4) [1]. *Aedes aegypti* and to a lesser degree, *Ae. albopictus* serve as vectors that transmit the dengue virus to the human body [1, 2]. An eight-fold increase over the last two decades, 5.2 million of dengue fever cases were reported in 2019 by the World Health Organization [3]. Despite being the most common vector-borne disease globally, the Western Pacific, Southeast Asia, and America are severely impacted by dengue infections, and Asia represents 70% of the global burden of dengue [3].

Solomon Islands (SI), a lower-middle-income nation with almost 1000 islands resided by less than a million inhabitants [4], is listed as 155 out of 190 countries in human development category [5]. Various disease outbreaks have occurred in SI and have become a serious epidemiologic challenge for the country in recent years [6]. The high burden of dengue fever can be attributed to unplanned urbanization, low sanitation practices, and lack of knowledge regarding dengue fever [7]. However, some studies have proposed that dengue fever transmission would not have been successful without favorable climate conditions [8].

The Intergovernmental Panel on Climate Change has projected intense extreme weather events in the future despite active climate mitigation efforts [9, 10]. These events may exacerbate the burden of dengue fever in the Asia–Pacific [11]. A study from China has reported that dengue fever incidence is significantly associated with extremely high temperatures and rainfall [8]. A similar study conducted in Taiwan has suggested that temperature, rainfall, and sunshine hours play important roles in dengue transmission [12].

Owing to regional complexity and proximity to the confluence of the inter-tropical convergence zone and South Pacific convergence zone, most of the big islands in SI, including Guadalcanal, experience high annual rainfall and low seasonality [13]. Rainfall in SI is highly correlated with the El Niño Southern Oscillation (ENSO) cycle [14]. ENSO can be explained as an anomaly in sea surface temperature in the Niño 3.4 region with three phases, El Niño (hotter/drier), La Niña (cooler/wetter), and neutral phases. The amount of rainfall could increase twofold during La Niña relative to El Niño in Guadalcanal [13]. Moreover, it has been proven that ENSO can impact human health [15].

Developing public health adaptation strategies are necessary to address the effects of climate change. With regard to dengue fever in SI, such actions require a better understanding of the association between climate hazards and disease incidence. Despite that the effects of climate variables on dengue fever [12] have been explored, a paucity of information related to how climate affects dengue fever in SI is inevitable because of health outcomes and meteorological data constraints. However, our study has successfully extracted dengue-like illness (DLI) incidence from the Ministry of Health and Medical Services (MHMS) SI between 2015 and 2018. Using the Solomon Islands Syndromic Surveillance Report (SISS), this study investigates the associations between temperature, cumulative rainfall, and ENSO on vector-borne disease risks of DLI in SI.

## Materials and methods

### Study area

SI is an archipelagic country of 997 islands stretching in south of the equator in the Western Pacific Ocean. According to the Solomon Islands National Statistics Office, in 2018, the country had a population of 667,044, and 34.64% resided in Honiara City and Guadalcanal Province, which are situated in the main island of Guadalcanal Island [4].

**Fig. 1:**
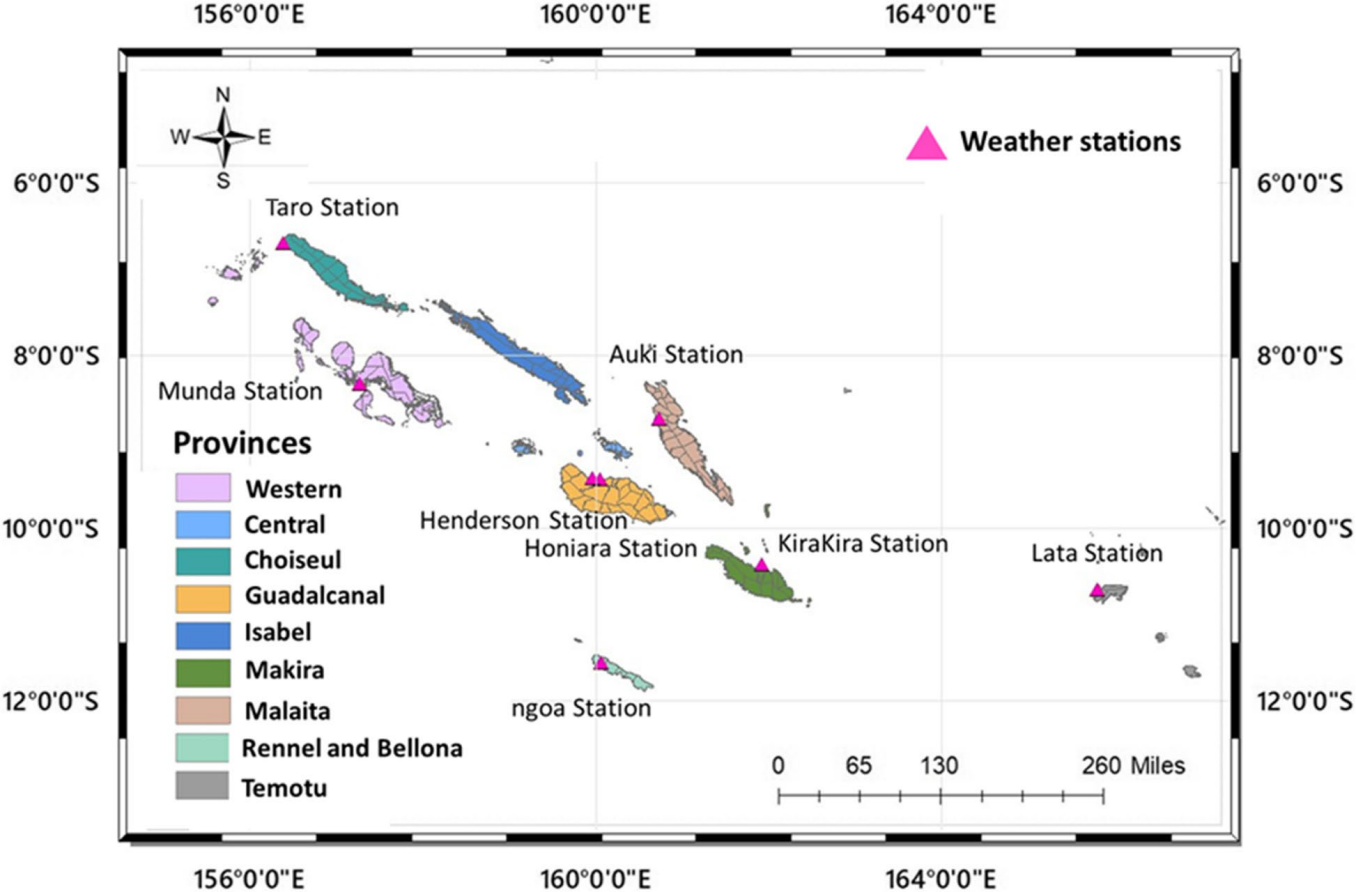
Location of study area

SI consists of one capital city and nine provinces, which are major islands distributed in the sea area and creating a long regional island chain: Guadalcanal; Malaita; Isabel; Western; Choiseul; Central; Rennell and Bellona; ​​Makira; and Temotu (Fig. 1). The capital, Honiara, is located on the largest island (Guadalcanal).

### Data sources

The weekly DLI data (2015–2018) of SISS were obtained from the Ministry of Health and Medical Services. The data were aggregated from weekly to monthly resolution. Province-level DLI data were used for analysis. The weather-DLI association analysis was assessed in two large provinces, Guadalcanal and Western Provinces, due to data availability. However, we provided the combined cases from the two provinces and labeled them as all Islands in our analysis.

A DLI case was defined according to the Practical Guide for Implementing Syndromic Surveillance in Pacific Island Countries and Territories since 2010, which includes fever (≥ 38 °C) for at least 2 days and two or more of the following symptoms: nausea/vomiting, muscle/joint pains, severe headache/pain behind the eyes, rash, bleeding, and negative malaria test [16]. A practical guide was made for the development of a simple and sustainable system that detects early unusual cases and clusters of infectious disease. The purpose of the system was to curb the impact of outbreaks.

We collected daily weather data, including maximum temperature, minimum temperature, and cumulative rainfall from weather observatories provided by Solomon Islands Government Ministry of Environment, Climate Change, Disaster Management and Meteorology (Fig. 1). The average temperature was obtained from the mean of maximum and minimum temperatures. We extracted information of monthly Oceanic Niño Index (ONI) values from NOAA, which records sea surface temperature anomalies in the Niño 3.14 region of the equatorial Pacific [17], to derive ENSO categories (El Niño, La Niña, and Neutral). Detailed information about ONI values is available online on NOAA Climate Prediction Center (https://origin.cpc.ncep.noaa.gov/).

The population data for each province were extracted from the Solomon Islands National Statistics Office, which provides open access to yearly population. Detailed information about population data is available on their official portal (https://www.statistics.gov.sb/).

### Statistical analysis

We aggregated weekly DLI cases and daily weather data to a monthly scale to ensure consistency of the temporal scale of the outcome and exposure variables with the ONI values. This study adopted a multivariate generalized estimating equation model with negative binomial regression [18] to evaluate the effects of temperature and rainfall on DLI incidence in SI. We evaluated the risks for Guadalcanal and Western Province, separately, and then we replicated it by summing the cases from both provinces. The following model is considered:

$$\mathbf{L}\mathbf{o}\mathbf{g}\left[\mathbf{Y}\right]\sim \left({{\varvec{t}}{\varvec{e}}{\varvec{m}}{\varvec{p}}{\varvec{e}}{\varvec{r}}{\varvec{a}}{\varvec{t}}{\varvec{u}}{\varvec{r}}{\varvec{e}}}^{\boldsymbol{*}},{\varvec{l}}{\varvec{a}}{\varvec{g}}\right)+\left({{\varvec{r}}{\varvec{a}}{\varvec{i}}{\varvec{n}}{\varvec{f}}{\varvec{a}}{\varvec{l}}{\varvec{l}}}^{\#},{\varvec{l}}{\varvec{a}}{\varvec{g}}\right)+\left(\mathbf{E}\mathbf{N}\mathbf{S}\mathbf{O}\ \mathbf{c}\mathbf{a}\mathbf{t}\mathbf{e}\mathbf{g}\mathbf{o}\mathbf{r}\mathbf{i}\mathbf{e}\mathbf{s}\right)+\left(\mathbf{t}\mathbf{i}\mathbf{m}\mathbf{e}\right)+\mathbf{o}\mathbf{f}\mathbf{f}\mathbf{s}\mathbf{e}\mathbf{t}\left(\mathbf{p}\mathbf{o}\mathbf{p}\mathbf{u}\mathbf{l}\mathbf{a}\mathbf{t}\mathbf{i}\mathbf{o}\mathbf{n}\right)$$

^*^ denotes for monthly average temperature

^#^ denotes for monthly cumulative rainfall

We conducted a multivariate analysis that included monthly average temperature, and cumulative rainfall with lag structures of up to two months (0–2 months) to capture the delayed effects of risk factors on the disease rates for the whole year (Model 1). The 2-month lag was selected to consider the mosquito’s lifespan and transmission cycle of the virus [19]. However, we also did the extension analysis with a longer lag of up to six months to see the possible effects of weather variables on DLI. The population statistics were included in the model as offset variables. We created dummy variables to indicate ENSO categories by labeling months with El Niño as “1”, Neutral as “2”, and La Niña as “3” and put it in the model. The selected predictors included in the final model were informed by the quasi information criterion (QIC) value of combined cases from the two provinces. We then performed additional assessment restricting the ONI values to El Niño (**≥ **+ 0.5), La Niña (≤ -0.5), and Neutral (> **-**0.5 – <  + 0.5) condition (Model 2). The risks from statistical analyses were reported as incidence rate ratios (IRRs) with 95% confidence interval (95% CI) and interpreted as risk every 1 °C increase of monthly average temperature and 10 mm increase of monthly cumulative rainfall. IRR has been widely used in epidemiological field to report the exposure of dependent variables on the risk of health outcomes [, 20, 21]. All analyses were computed using Stata/IC 15.0.

## Results

**Fig. 2:**
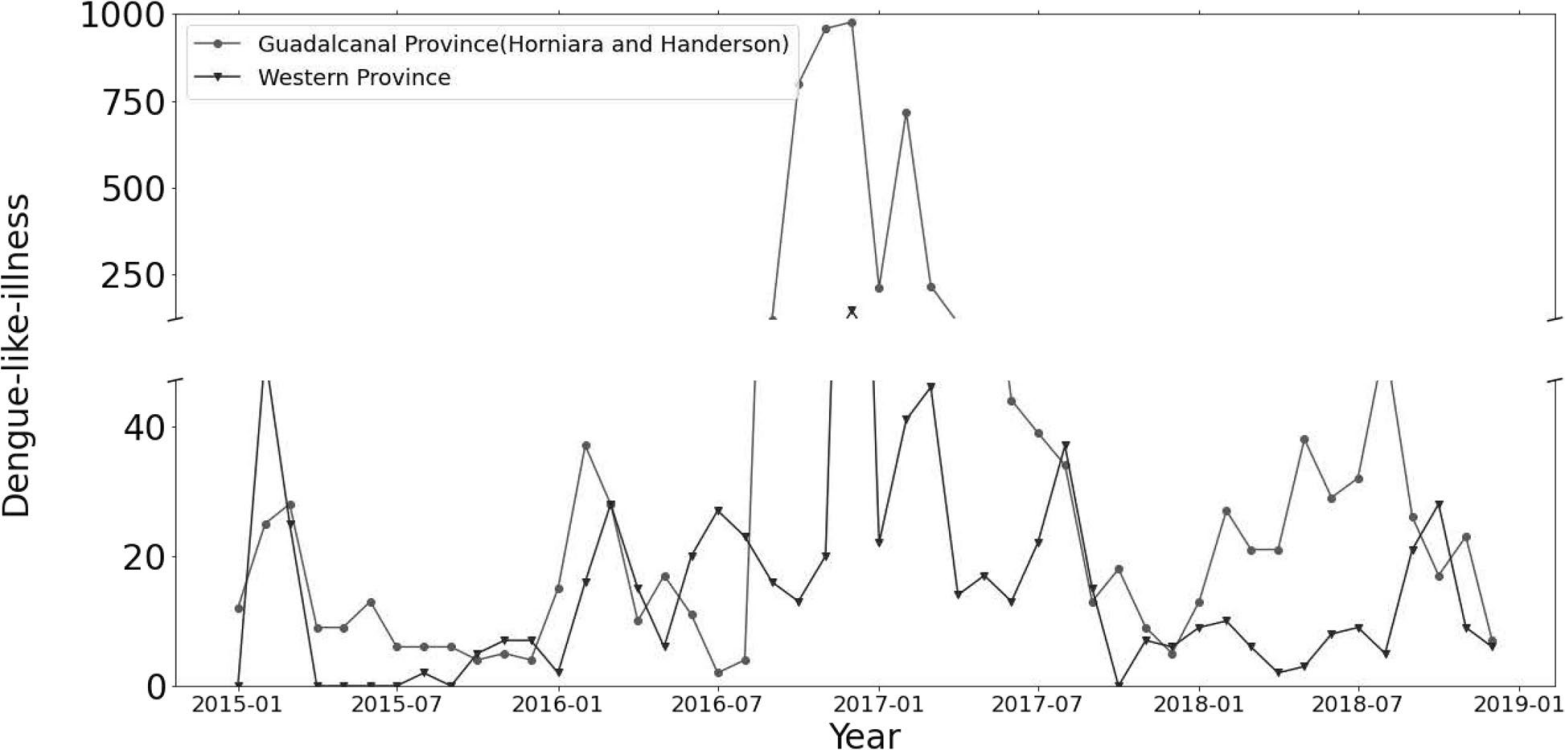
Monthly trend of dengue-like-ilness in Western and Guadalcanal Province of Solomon Islands from 2015 to 2018

**Fig. 3:**
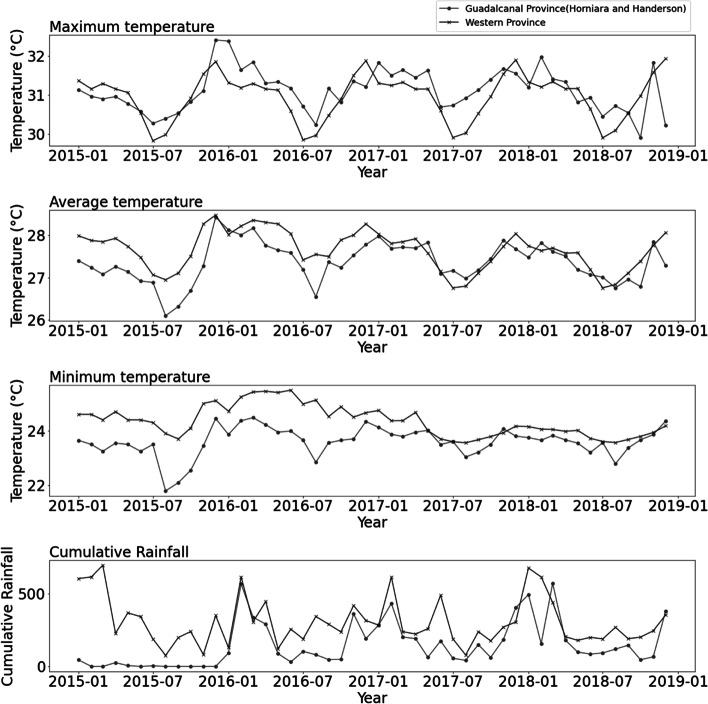
Trend of environmental factors from 2015 to 2018 in in Western and Guadalcanal Province of Solomon Islands

During the study period of 2015–2018, a total of 5,684 dengue fever cases were reported in Guadalcanal Province and Western Province, and 4,900 of which occurred in Guadalcanal Province (Table 1). The monthly mean DLI incidence was 103 cases for Guadalcanal Province and 17 cases for Western Province, with a higher number of DLI cases was reported during La Niña conditions. Figure 2 shows a long outbreak between August 2016 and April 2017, and the highest peak was recorded on December 2016 with 977 cases in Guadalcanal Province and 147 cases in Western Province. The monthly mean minimum, average and maximum temperature of SI during the study period were 23.98 °C, 27.52 °C and 31.04 °C, respectively, and the cumulative rainfall was 227.62 mm (Table 1). The temporal trends of monthly minimum, average and maximum temperature, and cumulative rainfall of Guadalcanal Province and Western Province are depicted in Fig. 3.

**Table 1:** Means and ranges of monthly dengue-like-illness and weather variables in Western and Guadalcanal Provinces of Solomon Island from 2015 to 2018

	Total cases	Mean	St. dev	Min	P25	P50	P75	Max
**Meteorological parameters**								
Maximum temperature		31.04	0.58	29.83	30.64	31.16	31.36	32.41
Minimum temperature		23.98	0.67	21.8	23.61	23.92	24.38	25.49
Average temperature		27.52	0.5	26.1	27.15	27.57	27.86	28.48
Cumulative rainfall		227.62	179.78	0	81.72	191.48	322.35	698.1
**Dengue cases**								
Guadalcanal Province	4,900	103	239	2	9	21	39	977
Western	784	17	23	0	5	10	21.25	145
**Dengue cases during El Niño (** ***n*** ** = 16 months)**								
Guadalcanal Province	199	12	9	4	6	9	16	37
Western	125	8	9	0	0	6	12	28
**Dengue cases during neutral (** ***n*** ** = 22 months)**								
Guadalcanal Province	1,756	80	154	2	17	31	53	717
Western	427	20	15	0	8	19	25	51
**Dengue cases during La Niña (** ***n*** ** = 10 months)**								
Guadalcanal Province	2,945	295	430	5	13	24	798	977
Western	232	24	44	0	6	10	16	145

*Note*: P25, P50 and P75 represent the measurements at 25^th^, 50^th^, and 25^th^ percentiles

Monthly average temperature, cumulative rainfall, and ENSO categories were included in the model for the estimation of the risk of DLI in SI according to the QIC values of combined cases from the two provinces (Supplementary Table S1). Table 2 shows the lagged effects of monthly average temperature, cumulative rainfall, and the risk of DLI in SI. An increase of 1 °C monthly average temperature was associated with an increased risk of DLI in All Islands and Guadalcanal Province, and the highest risk was found in Guadalcanal Province at lag 1 month (IRR: 2.186, 95% CI: 1.094–4.368). This study did not observe significant effects on Western Province. However, monthly average temperature had no significant effect on DLI risk after lag 2 months (Supplementary Table S2).

**Table 2:** The lag specific up to two months incidence rate ratios (95% confidence interval) depicting association between meteorological variables on dengue-like-illness adjusting each other by controlling ENSO category in Guadalcanal and Western Province from 2015 to 2018

	Lag 0	Lag 1	Lag 2
Guadalcanal Province			
Avg. temperature	1.618 (0.813–3.219)	**2.186 (1.094–4.368)**	1.956 (0.968–3.954)
Rainfall	**1.003 (1.001–1.005)**	1.002 (0.999–1.004)	0.999 (0.998–1.002)
Western Province			
Avg. temperature	1.464 (0.733–2.925)	1.283 (0.639–2.572)	1.549 (0.776–3.094)
Rainfall	**1.001 (1.001–1.003)**	**1.002 (1.001–1.003)**	0.999 (0.998–1.002)
All Islands^a^			
Avg. temperature	**1.748 (1.107–2.758)**	**1.973 (1.237–3.147)**	1.606 (0.996–2.588)
Rainfall	**1.002 (1.001–1.004)**	**1.001 (1.000–1.002)**	0.999 (0.998–1.001)

^a^All Islands: combined cases number from those province

This study found that population of SI was vulnerable to rainfall. A minor effect of cumulative rainfall was captured, with 0.001%–0.003% increase in risk every 10 mm increase of monthly cumulative rainfall, which was consistent across 2 Islands (Table 2). The highest risk of cumulative rainfall was found in Guadalcanal Province at lag 1 month, with IRR of 1.003 (95% CI: 1.001–1.005). The effects of cumulative rainfall disappeared after lag zero in Guadalcanal and one month in Western Province and All Islands. Our results show there was no significant association between cumulative rainfall and DLI risk in SI when we extended the lag up to six months (Supplementary Table S2).

**Fig. 4:**
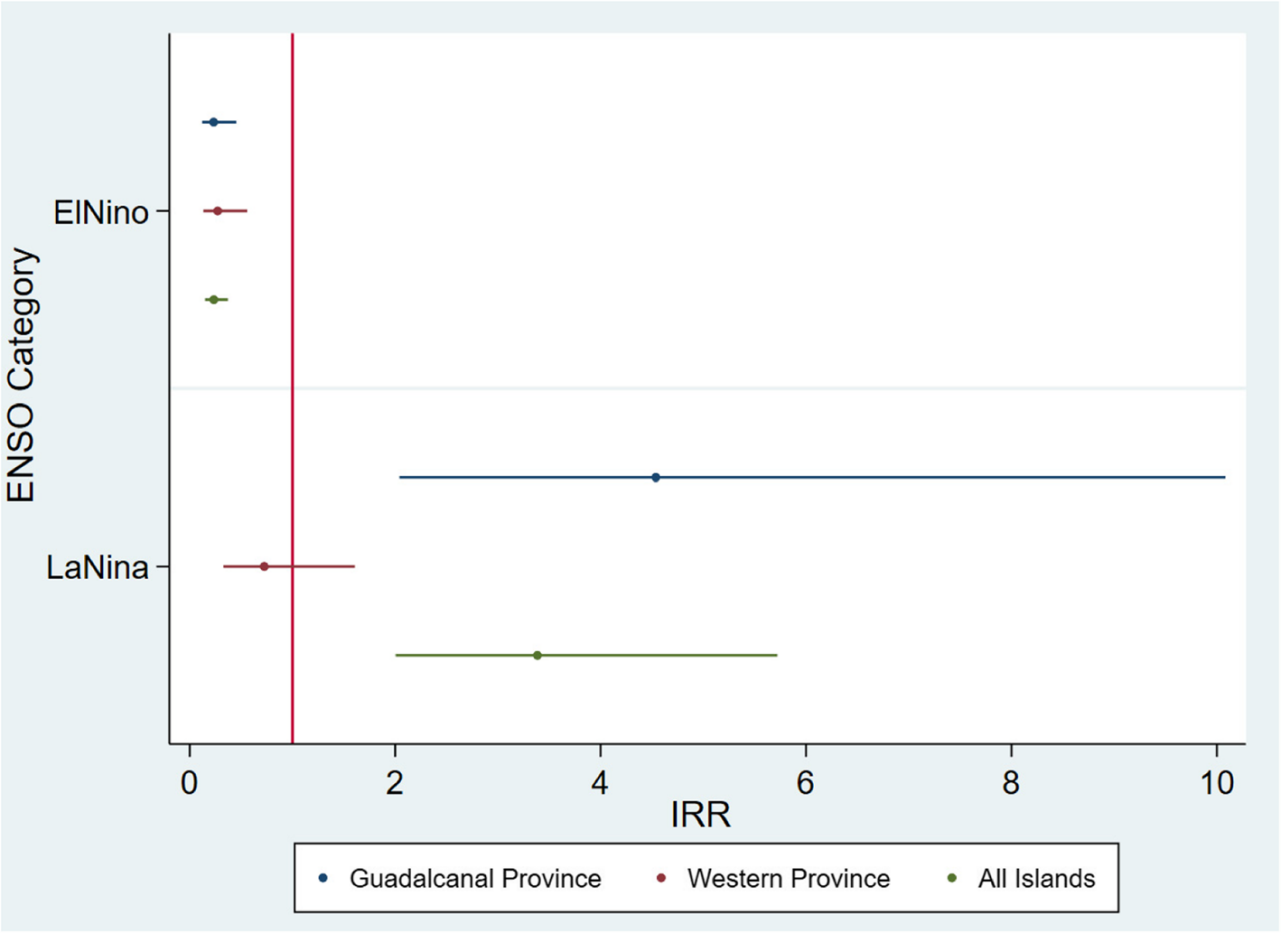
IRR and 95% CI for ENSO category (El Niño and La Niña, vs. neutral) and risk of dengue-like-illness by controlling temperature and precipitation in Western and Guadalcanal Province of Solomon Island from 2015–2018 (All Islands represents the combined cases number from provinces)

To further investigate the role of ONI index on DLI, we conducted a sub-analysis restricted to the ENSO category only (Fig. 4 and Supplementary Table S3). Compared to the ENSO neutral category, El Niño category consistently reduced the risk of DLI in the studied area, with reductions ranging from 72.8% to 76.7%. On the other hand, La Niña greatly increased the risk of DLI, and the highest association was found in Guadalcanal Province (IRR: 4.537, 95% CI: 2.042–10.083) and All Islands category (IRR: 3.386, 95% CI: 2.004–5.722). No positive association effects were found in Western Province.

## Discussion

Climate is one of the overriding factors of the current dengue incidence situation and should not be underestimated [12]. To the best of our knowledge, evidence of the association between climatic factors and ENSO on DLI incidence in the Western Pacific region, especially SI, is lacking. Therefore, this study evaluated the effects of temperature, rainfall, and ENSO (La Niña and El Niño) on the burden of DLI in SI. Our data capture a long dengue outbreak between 2016 and early 2017 with the highest peak on December 2016. The outbreak was noted with uncommon increase of DLI along with the confirmed cases of dengue in the Solomon Islands. A group of researchers identified that the massive outbreak in 2016–17 was mostly caused by DENV-2 [6].

Our data were collected onsite from SI bureaus to conduct the analysis. In this study, we found that temperature had a significant effect on the risk of DLI in Guadalcanal Province and rainfall showed minor yet consistent effects on the islands. Moreover, the risk of La Niña on DLI was remarkably high in Guadalcanal Province, and protective effects were observed during the El Niño period in SI.

Temperature is one of the most important climatic factors in dengue transmission. Temperature influences the life cycle (gonotrophic cycle, growth, and development rate) of mosquitoes and the development of pathogens inside a vector [22]. Warm temperatures can accelerate viral replication, shorten extrinsic incubation time and induce the biting rates of mosquitos [23]. An Indonesian study reported that temperature has a significant correlation with dengue hemorrhagic fever (*r* = 0.371,*p* = 0.040) [24]. Likewise, our study found that temperature was associated with increased risk of DLI in Guadalcanal Province.

The duration and quantity of rainfall are important to dengue transmission. Rainfall can provide favorable habitats for aquatic stages (e.g., larvae) and enhance their distribution [25, 26]. This situation may apply to *Ae. albopictus* to a greater extent rather than *Ae. aegypti,* which is better known to breed indoors [27]. A study in a tropical city-state of Singapore has shown that high rainfall has a significant effect on dengue transmission after 5 weeks [28]. A study in Malaysia confirmed that accumulated rainfall would affect the dengue cases at a cumulative change of 21.45% (95% CI: 8.96, 51.37) with the highest effect delayed by 26–28 days [29]. Likewise, a Taiwan study has reported that high rainfall can lead to an increase in dengue incidence at a specific time (lag 10-weeks) [25]. Meanwhile, excessive rainfall can flush mosquitoes’ habitats in the short-term periods (< 5 weeks), disrupting the survival and transmission of the dengue virus [25]. The plausible explanation to the lagged effect can be explained because of the incubation periods in hosts, including the aquatic and aerial stages of mosquitoes. In line with our study, we observed a positive relationship between rainfall and an increment in DLI risk across all regions. Our study indicated that the risk of rainfall should not be neglected in SI.

To date, ENSO is linked with extreme weather events (floods and drought) and is known to exacerbate vector-borne disease risks in areas where climate is associated with the ENSO cycle (2–7 years), and disease prevention is insufficient [15, 30]. We observed that while DLI risks increased in Guadalcanal Province during the La Niña phase, the effects of El Niño varied between the two provinces. The nebulous relationship between dengue and La Niña is exemplified in our results by the positive association of dengue risks with precipitation and La Niña phase. Overall, SI experiences colder and higher precipitation during La Niña phase [14].

As an archipelagic nation, SI has mountainous regions as well as low-lying atolls scattered across the country. Meteorological Services Division in SI reported that the temperature tends to be uniform (very less seasonality) in most areas throughout the year, with a mean maximum temperature of approximately 2 °C. The annual rainfall is between 3000 and 5000 mm, and the greatest annual rainfall can reach up to 9000 mm at some elevated locations (Ministry of Environment [31). A report suggested that the SI rainfall historical data has an upward trend, whereas the annual rainfall is expected to increase with the intensity of extreme rainfall days in the islands [32]. Projected alteration to rainfall patterns will not necessarily create new threats to health sectors but will likely exacerbate the current situation. The government is encouraged to enhance measurement strategies to control mosquito breeding and the distributions and intensities of other infectious disease vectors.

Although we have established insightful findings regarding the effects of climatic conditions on DLI in SI, this study has some limitations. First, owing to the paucity of long-term data, the empirical estimates of climatic effects on DLI may vary when more data are available. Second, climate condition is not the only risk factor for vector-borne diseases. Our study did not consider the present seroprevalence, herd immunity, secondary transmission, socioeconomic factors, environment and displacement of dengue serotypes. Secondary transmission is a complex situation, which is related to vector control, travel history, and dengue stereotype [33]. Furthermore, surveillance data defined the DLI according to the clinical presentation, and dengue fever can be asymptomatic [34]. Thus, unreported cases can underestimate the true extent of climate variability on the risk of DLI. A previous study conducted in the tropical city-state of Singapore has estimated that asymptomatic cases can be 19-fold [34].

Additionally, our research is an ecological study, and thus our findings cannot be generalized at the individual level. However, the results are based on the best available data collected from SI government bureaus. The number of dengue cases and outbreaks have increased dramatically in SI; therefore, a seasonal forecasting system that can predict DLI incidence should be developed. Our study has offered information for decision-makers to initiate strong preventive health policies that support active dengue surveillance and monitor breeding sources. More information is recommended for the development of robust early warning systems.

## Conclusion

In terms of climate change, efforts to strengthen public health interventions for controlling dengue transmission are needed to curb the number of dengue outbreaks. The dengue outbreaks in 2016 and 2017 imposed serious public health burden on the Western Pacific, especially in SI. This study reveals the potential transmission of DLI as a result of maximum temperature and rainfall. The average temperature was significantly associated with the risk of DLI incidence. In addition, precipitation and La Niña significantly affected the risk of DLI in SI. Therefore, integrating climatic variability into dengue intervention programs will help decision-makers establish a sustainable and strong public health adaptation strategies against climate changes.

## Supplementary Information

**Additional file 1: Supplementary Table S1.** Model configuration based on Quasi Information Criterion (QIC). **Supplementary Table S2.** The lag specific up to six months incident rate ratios (95% confidence interval) depicting association between meteorological variables on dengue-like-illness adjusting each other by controlling ENSO category with the Quasi Information Criterion (QIC) in Guadalcanal and Western Province from 2015 to 2018. **Supplementary Table S3.** The incident rate ratios (95% confidence interval) depicting association between ENSO category (El Niño and La Niña, vs. neutral) on dengue-like-illness by controlling temperature and precipitation in Guadalcanal and Western Province from 2015 to 2018.

## Data Availability

Data not available due to [ethical/legal/commercial] restrictions but are available from the corresponding author on reasonable request.
