# Supplementary material for: Effects of temperature, rainfall, and El Niño Southern Oscillations on dengue-like-illness incidence in Solomon Islands
Source: BMC Infect Dis. 2023 Apr 6;23:206. doi: 10.1186/s12879-023-08188-x (PMC10080901; doi:10.1186/s12879-023-08188-x)
Supplement: Supplementary file 1 — Additional file 1: Supplementary Table S1. Model configuration based on Quasi Information Criterion (QIC). Supplementary Table S2. The lag specific up to six months incident rate ratios (95% confidence interval) depicting association between meteorological variables on dengue-like-illness adjusting each other by controlling ENSO category with the Quasi Information Criterion (QIC) in Guadalcanal and Western Province from 2015 to 2018. Supplementary Table S3. The incident rate ratios (95% confidence interval) depicting association between ENSO category (El Niño and La Niña, vs. neutral) on dengue-like-illness by controlling temperature and precipitation in Guadalcanal and Western Province from 2015 to 2018. [file 12879_2023_8188_MOESM1_ESM.docx]

Supplementary Table S1. Model configuration based on Quasi Information Criterion (QIC)

| Model configuration | QIC |
| --- | --- |
| Average temperature | 1042 |
| Rainfall | 932 |
| Average temperature + rainfall | 1056 |
| **Average temperature + Rainfall + ENSO categories*** | **928** |

QIC: Quasi information criterion

* Best-fit model: The model is adjusted by population

Supplementary Table S2. The lag specific up to six months incident rate ratios (95% confidence interval) depicting association between meteorological variables on dengue-like-illness adjusting each other by controlling ENSO category with the Quasi Information Criterion (QIC) in Guadalcanal and Western Province from 2015 to 2018

|  | Lag 0 | Lag 1 | Lag 2 | Lag 3 | Lag 4 | Lag 5 | Lag 6 |
| --- | --- | --- | --- | --- | --- | --- | --- |
| Guadalcanal Province | | | | | | | |
| Tavg | 1.618 (0.813-3.219) | **2.186 (1.094-4.368)** | 1.956 (0.968-3.954) | 0.983 (0.483-2.001) | 0.602 (0.293-1.239) | 0.395 (0.185-0.844) | 1.479 (0.689-3.173) |
| Rainfall | **1.003 (1.001-1.005)** | 1.002 (0.999-1.004) | 0.999 (0.998-1.002) | 1.001 (0.999-1.003) | 0.999 (0.997-1.001) | 0.999 (0.997-1.001) | 0.999 (0.997-1.001) |
| Western Province | | | | | | | |
| tavg | 1.464 (0.733-2.925) | 1.283 (0.64-2.572) | 1.549 (0.776-3.094) | 1.681 (0.841-3.360) | 2.006 (0.997-4.037) | 1.392 (0.676-2.865) | 0.358 (0.213-0.601) |
| Rainfall | **1.001 (1.001-1.003)** | **1.002 (1.001-1.003)** | 0.999 (0.998-1.002) | 0.998 (0.996-1.000) | 0.998 (0.996-1.000) | 0.999 (0.997-1.001) | 0.998 (0.997-0.999) |
| All Islands | | | | | | | |
| tavg | **1.748 (1.107-2.758)** | **1.973 (1.237-3.147)** | 1.606 (0.996-2.588) | 1.071 (0.661-1.738) | 0.957 (0.585-1.564) | 0.681 (0.408-1.137) | 0.237 (0.109-0.517) |
| Rainfall | **1.002 (1.001-1.004)** | **1.001 (1.000-1.002)** | 0.999 (0.998-1.001) | 1.000 (0.998-1.001) | 0.998 (0.996-0.999) | 0.998 (0.996-0.999) | 1.000 (0.998-1.002) |
| QIC | 928 | 859 | 838 | 823 | 795 | 798 | 812 |

Supplementary Table S3. The incident rate ratios (95% confidence interval) depicting association between ENSO category (El Niño and La Niña, vs. neutral) on dengue-like-illness by controlling temperature and precipitation in Guadalcanal and Western Province from 2015 to 2018

|  | Guadalcanal Province | Western Province | All Islands* |
| --- | --- | --- | --- |
| ENSO |  |  |  |
| Neutral | - | - | - |
| El Niño | 0.233 (0.119-0.454) | 0.272 (0.132-0.559) | 0.235 (0.149-0.372) |
| La Niña | **4.537 (2.042-10.083)** | 0.726 (0.328-1.607) | **3.386 (2.004-5.722)** |

*All Islands: combined cases number from those provinces
